# Detecting Class 1 Integrons and Their Variable Regions in *Escherichia coli* Whole-Genome Sequences Reported from Andean Community Countries

**DOI:** 10.3390/antibiotics13050394

**Published:** 2024-04-25

**Authors:** María Nicole Solis, Karen Loaiza, Lilibeth Torres-Elizalde, Ivan Mina, Miroslava Anna Šefcová, Marco Larrea-Álvarez

**Affiliations:** 1 Facultad de Ciencias Médicas Enrique Ortega Moreira, Carrera de Medicina, Universidad Espíritu Santo, Samborondón 092301, Ecuador; 2 Department of Bacteria, Parasites and Fungi, Statens Serum Institut, 2300 Copenhagen, Denmark; 3 Graduate School Life Sciences and Health (GS LSH), Université Paris-Saclay, 91198 Gif-sur-Yvette, France; 4 School of Biological Science and Engineering, Yachay-Tech University, Urcuquí 100650, Ecuador

**Keywords:** *Escherichia coli*, class 1 integrons, *dfrA* genes, *aadA* genes, bioinformatic tools, Andean Community

## Abstract

Various genetic elements, including integrons, are known to contribute to the development of antimicrobial resistance. Class 1 integrons have been identified in *E. coli* isolates and are associated with multidrug resistance in countries of the Andean Community. However, detailed information on the gene cassettes located on the variable regions of integrons is lacking. Here, we investigated the presence and diversity of class 1 integrons, using an in silico approach, in 2533 whole-genome sequences obtained from EnteroBase. IntFinder v1.0 revealed that almost one-third of isolates contained these platforms. Integron-bearing isolates were associated with environmental, food, human, and animal origins reported from all countries under scrutiny. Moreover, they were identified in clones known for their pathogenicity or multidrug resistance. Integrons carried cassettes associated with aminoglycoside (*aadA*), trimethoprim (*dfrA*), cephalosporin (*blaOXA*; *blaDHA*), and fluoroquinolone (*aac(6′)-Ib-cr*; *qnrB*) resistance. These platforms showed higher diversity and larger numbers than previously reported. Moreover, integrons carrying more than three cassettes in their variable regions were determined. Monitoring the prevalence and diversity of genetic elements is necessary for recognizing emergent patterns of resistance in pathogenic bacteria, especially in countries where various factors are recognized to favor the selection of resistant microorganisms.

## 1. Introduction

*Escherichia coli* is widely distributed in natural environments and is a common inhabitant of the gastrointestinal tract in animals, including humans. While it serves as an important commensal resident, it also poses a threat as a potential pathogen, capable of causing both intestinal and extra-intestinal diseases [1,2]. The rise of multidrug-resistant (MDR) *E. coli* can be attributed to prolonged antibiotic exposure, presenting substantial hurdles for public health systems [3]. This phenomenon has been observed globally, with prevalence rates influenced by geographical regions, populations, and countries [4,5]. In South America, several factors contribute to the dissemination of antimicrobial resistance. These include drug misuse in the community, the spread of genetic markers through the food chain, and environmental contamination from sewage disposal, not only from hospitals but also from industrial and urban sources [6]. In particular, strains of *E. coli* resistant to commonly used antibiotics have been documented in countries comprising the Andean Community—an intergovernmental organization consisting of Bolivia, Colombia, Ecuador, and Peru [7,8,9,10].

Genetic changes and the horizontal transmission of resistant traits are recognized as crucial factors driving the development of antimicrobial resistance. MDR phenotypes arise as bacteria acquire and disseminate resistance genes through horizontal transfer [11,12]. This process is facilitated by genetic elements such as gene cassettes/integrons, insertion sequences (IS), and transposons, which have the ability to move between and within DNA molecules. Additionally, plasmids and conjugative elements play a role in mobilizing genetic information between bacterial cells [13]. Integrons, in particular, are capable of integrating gene cassettes through natural recombination events, making them adequate platforms for gene expression [14]. They are associated with IS, transposons, and plasmids, which collectively facilitate the spread of resistance traits among bacterial populations [15,16]. A typical integron consists of a variable region and two conserved segments. These segments are known as the 5′ and 3′ conserved sequences (CS). The *intI1* gene is situated within the 5′ CS and encodes a tyrosine recombinase responsible for integrating or excising genes at the *attI* site. Additionally, the *intI1* gene contains the (Pc) promoter sequence, which drives the expression of gene cassettes located within the variable region [17,18]. Integrons are classified into five groups based on the sequence of the *intI1* gene they carry, with class 1 integrons being particularly prevalent and clinically significant [15,16]. In contrast, the 3′ CS region may incorporate genes conferring resistance to sulphonamides (*sul1*) or quaternary ammonium compounds (*qacEΔ1*) [17]. These genes are frequently encountered in class 1 integrons, while class 2 integrons often harbor genes associated with transposition proteins (tns). Other classes of integrons have not been associated with gene cassettes [15]. The variable region of the integron, situated between these two segments, serves as a platform for the integration of various cassettes that confer resistance to multiple classes of antibiotics [15]. These gene cassettes typically consist of an open reading frame and a recombination site (*attC*), and they are integrated at the *attI* site and expressed from the Pc promoter. The level of transcription depends on the proximity of the cassette to the promoter, with genes located closer to it exhibiting higher expression levels [18]. Class 1 integrons have been linked to a range of gene cassettes conferring resistance to aminoglycosides, folate antagonists, β-lactams, quinolones, and other classes of antibiotics [16]. 

These integrons are frequently encountered in enteropathogenic bacteria [16]. *Escherichia*, *Klebsiella*, *Salmonella*, *Shigella*, and *Yersinia* are notable genera of the *Enterobacteriaceae* family that colonize the intestinal tract and can lead to intestinal, genitourinary, and bloodstream infections [19]. As mentioned earlier, the emergence of MDR *E. coli* has been linked to prolonged exposure to antibiotics. Indeed, class 1 integrons have been identified in *E. coli* isolates from various geographical regions [20]. The prevalence of these platforms and their associated cassettes is known to fluctuate over time, a phenomenon linked to selective antibiotic pressure. An increase in the prevalence of class 1 integrons in *E. coli* has been observed in South Korea and China [16,20]. Moreover, the incidence of class 1 integrons harboring multiple cassettes has increased among *E. coli* isolates, suggesting that they facilitate the acquisition of gene cassettes [16,20]. Therefore, the continuous monitoring of integrons is crucial, particularly for understanding the spread of drug resistance. 

As mentioned previously, strains of MDR *E. coli* have been documented in countries forming the trade block called the Andean Community [7,8,9,10]. This organization promotes collaboration in industry, agriculture, social issues, and trade. The constant flow of people and resources increases the risk of the cross-transmission of microorganisms, particularly those resistant to multiple drugs. Integrons have been identified in MDR and pathogenic isolates of *E. coli* belonging to various clones reported in the countries under scrutiny. Several studies have documented the presence of various antibiotic-resistant genes, primarily *dfrA* and *aadA*, in the variable regions of these integrons [21,22,23,24], while others have solely documented the presence of the *intI1* gene [25,26,27]. Class 1 integrons have been linked to an increase in MDR *E. coli*, attributed to the diversity of cassettes within their variable regions [20,28]. The limited information available on these cassettes among isolates from Andean countries may hinder our ability to study their dynamics and their relationship with resistance genes. 

Bioinformatic tools have not only facilitated the identification of mobile genetic elements but have also helped establish crucial connections between resistance traits and pathogenic bacteria [29,30]. The objective of this study is to use IntFinder v1.0, a tool capable of detecting integrons in both raw reads and assembled genomes [31,32], to identify the association of integrons with resistance cassettes in isolates from countries within the Andean Community.

## 2. Results

### 2.1. Bacterial Isolate Dataset

The final dataset comprised 2533 whole-genome sequenced isolates of *E. coli* documented in countries of the Andean Community (Table S1, Supplementary Materials). The majority of isolates were reported in Ecuador (75%), followed by Peru (19%), Colombia (3%), Venezuela (2%), and Bolivia (1%). Nearly half of the isolates were sourced from human samples (47%), followed by those from animal samples (31%), the environment (20%), and food products (2%).

### 2.2. Integron Characterization by Country and Source

**Figure 1:**
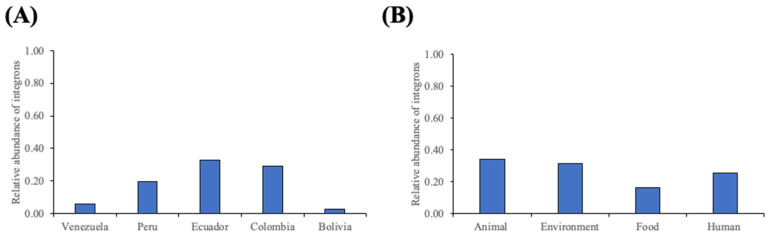
Abundance of integrons detected in *E. coli* isolates classified by (**A**) country and (**B**) source.

IntFinder v1.0 identified integrons in 29% of isolates, all classified as class 1 integrons (Table S2, Supplementary Materials). These integrons were distributed across all countries, with the highest prevalence observed in samples from Ecuador, followed by Peru, Colombia, Venezuela, and Bolivia. However, in terms of relative abundance, approximately 30% of isolates from Ecuador and Colombia tested positive for integrons, while they accounted for 20% in Peruvian samples. Less than 1% of isolates from Venezuela and Bolivia showed evidence of integrons (Figure 1A). These class 1 integrons were detected across various sources, including human, animal, environmental, and food-related samples. However, analysis of the relative abundance revealed that integrons were present in approximately 30% of environmental and animal samples, while 25% of human samples carried these sequences. Class 1 integrons were found in less than 20% of isolates derived from food or environmental samples (Figure 1B).

### 2.3. Integron Characterization by ST

**Figure 2:**
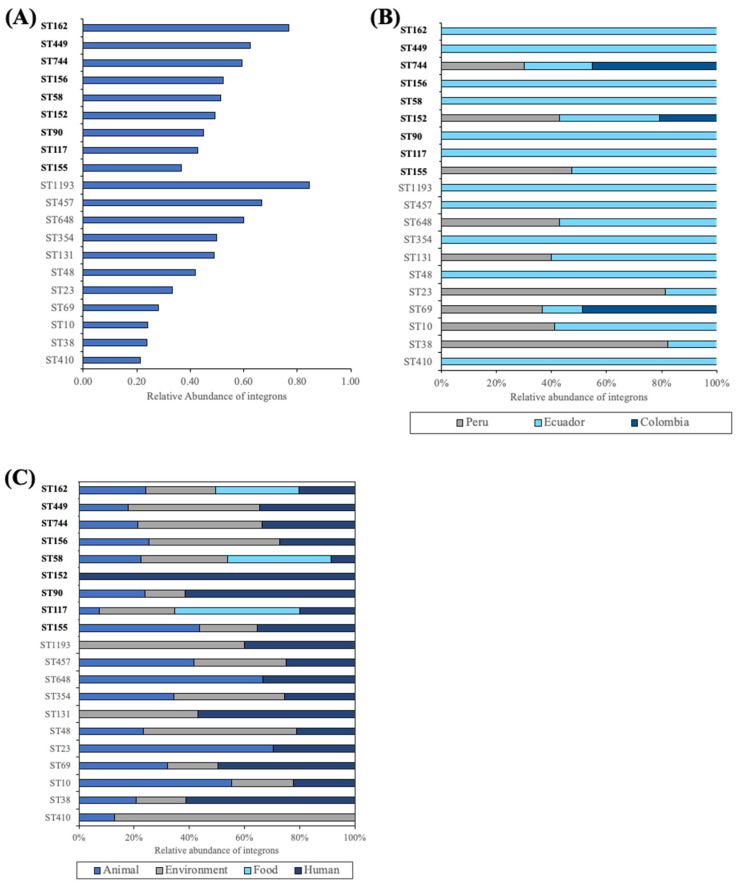
Abundance of integrons detected in *E. coli* isolates according to sequence type (**A**), organized by (**B**) country and (**C**) source. MDR STs are highlighted in bold.

Integron-positive isolates were associated with 149 different sequence types (STs). Figure 2A illustrates the prevalence of integrons among isolates associated with relevant pathogenic or MDR STs; the latter are highlighted in bold in the figure. In MDR clones, the highest occurrence of integrons was observed in isolates from ST162 (77%), followed by those associated with ST449 (63%) and ST744 (59%). Approximately half of the isolates belonging to ST156, ST58, and ST152 were positive for integrons, while in *E. coli* from ST90, ST117, and ST155, integrons were present in less than 50% of isolates. In pathogenic *E. coli*, integrons were most abundant in bacteria from ST1193 (85%), ST457 (67%), and ST648 (60%), whereas, in isolates from ST354 and ST131, integrons were present in half of them. They were also detected in 40% and 33% of *E. coli* associated with ST48 and ST23, respectively. In bacteria belonging to the remaining STs, these platforms were present in less than 30% of isolates.

Figure 2B,C depicts the aforementioned sequence types categorized by country and source, respectively. Integron-containing *E. coli* isolates belonging to STs known for multidrug resistance were predominantly reported in Ecuador, although bacteria from Peru were also associated with ST155, ST744, and ST152. *E. coli* from the latter two STs were also found in Colombian samples. Isolates from these STs mainly originated from environmental, human, and animal sources. ST117, ST58, and ST162 were also linked to food samples, while ST152 and ST156 were found in human samples. On the other hand, STs associated with pathogenic bacteria were predominantly documented in Ecuador, although some isolates belonging to ST648, ST131, ST23, ST69, ST10, and ST38 were also reported in Peru. *E. coli* from ST69 was also found in Colombia. Most of the STs were linked to animal, environmental, and human samples. Bacteria from ST1193 and ST131 originated from human and environmental samples, whereas those of ST648 and ST23 came from animal and human samples.

### 2.4. Integrons and Antibiotic Resistance Genes

**Figure 3:**
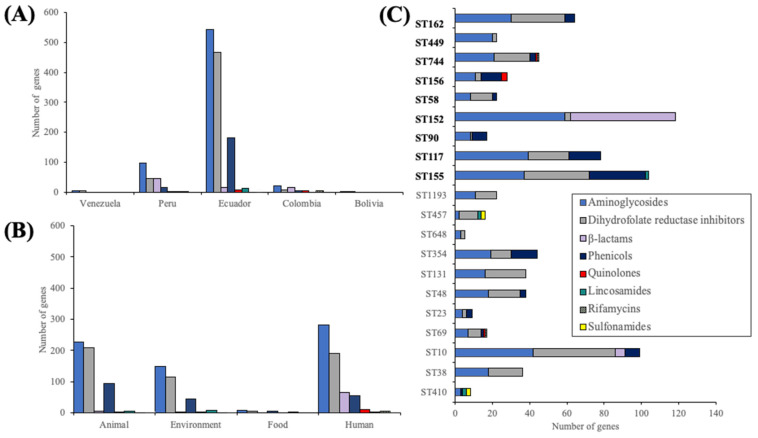
Integron-associated resistance genes classified by (**A**) country, (**B**) source of isolation, and (**C**) sequence type.

Gene cassettes conferring resistance to aminoglycosides and dihydrofolate reductase inhibitors were the most prevalent, were detected across samples from all sources reported in all five countries, and were associated with all the aforementioned STs. Similarly, genes conferring resistance to phenicols were identified in isolates from Ecuador and Peru. Integrons carrying these genes were reported from various sources. Conversely, genes associated with β-lactam resistance were primarily detected in human isolates reported from Peru, Ecuador, and Colombia. Resistance markers for lincosamide and sulfonamide antibiotics were found in environmental and animal samples, with the latter also associated with genes conferring resistance to quinolones. These markers were also detected in human isolates, along with those associated with rifampicin resistance. All STs exhibited resistance markers for aminoglycosides and folate antagonists. Additionally, resistance to chloramphenicol was observed across most of them. Genes encoding β-lactam hydrolases were prevalent, particularly in ST152 and ST10. Among the ST156, ST744, and ST69 clones, there were also traits associated with quinolone resistance. Furthermore, rifampicin-resistant genes were detected in the latter two STs (Figure 3).

A total of 37 different integrons were identified by IntFinder v1.0. Resistance to aminoglycosides was mediated by adenylyl transferases encoded by seven different *aadA* alleles, while resistance to trimethoprim was associated with dihydrofolate reductases encoded by seven different *dfrA* alleles. Inactivation of aminoglycosides was also facilitated by drug-modifying enzymes, including adenylyl and acetyl transferases encoded by the *ant(2′)* and *aac(3)-Vla* genes, respectively. Additionally, resistance was linked to the *aac(6′)-lb-cr* gene, identified in certain integrons, encoding an acetyl transferase capable of inactivating both aminoglycoside and quinolone antibiotics. Quinolone resistance was also associated with the *qnrB* genes, which produce proteins that bind to topoisomerases and protect them from drugs. β-lactam inactivation was facilitated by hydrolases encoded by the *blaOXA* and *blaDHA* genes, while resistance to rifampicin was attributed to enzymes encoded by the *arr* genes, catalyzing the ADP-ribosylative inactivation of the antibiotic (Table 1).

Resistance to clindamycin was linked to nucleotidyl transferases encoded by the *lnu(F)* genes. Similarly, sequences associated with transferases that inactivate chloramphenicol (*catB*) were identified, although resistance to this antibiotic was mainly attributed to efflux pumps encoded by the *cmlA1* genes. Additionally, *sul3* genes were found, responsible for conferring resistance to sulfonamide antibiotics by encoding dihydropteroate synthases. Regarding the variable regions, approximately half of the integrons harbored an *aadA* cassette in the first position, while 30% contained a *dfrA* cassette. Among the remaining 20% of isolates, *aac* (8%), *bla* (6%), *ant* (3%), and *lnu* (3%) genes were identified as the first cassette. The majority of positive isolates contained integrons with either one of two cassettes in the variable regions. The predominant genes identified were *aadA* and *dfrA*, along with additional genes encoding β-lactamases, acetyl transferases, and nucleotidyl transferases. Integrons with three cassettes were detected in 16% of isolates, while those containing four cassettes represented 11% of the samples. Only a small fraction, less than 1% of isolates, exhibited a single integron with more than four cassettes in the variable region (Table 1).

## 3. Discussion

This study explores the presence and diversity of class 1 integrons among isolates documented in Ecuador, Colombia, Peru, Bolivia, and Venezuela, representing the Andean Community. Integrons were found in only 29% of isolates, displaying limited diversity in gene cassette content, albeit higher than previously reported. Despite this, the detected integrons contained genes conferring resistance to commonly used antibiotics, contributing to the emergence of multidrug-resistant phenotypes in *E. coli*. The use of bioinformatic tools has been instrumental in identifying mobile genetic elements carrying resistance genes and elucidating their relationship with pathogenic bacteria [29,30]. IntFinder v1.0, specifically, facilitates the detection of integrons using both raw reads and assembled genomes/contigs [31].

Integrons were found to be more common in isolates from Ecuador, Colombia, and Peru compared with those reported in Bolivia and Venezuela. Interestingly, integrons were equally distributed among animal, environmental, and human samples, but they were less frequently identified in those obtained from food samples. In Ecuador, integrons were detected in human, animal, environmental, and food sources, consistent with findings from previous studies [22,24,33,34]. In Peruvian isolates, integrons were detected in samples from both humans and animals, while Colombian isolates showed an association between these platforms and clinical sources. Indeed, integrons have been reported in *E. coli* from patients and farm animals [10,21,35], although there are limited data available on their presence in environmental samples. Clinical isolates from Bolivia and Venezuela were associated with integrons, although they have also been reported in environmental samples [36,37].

ST162 is recognized as a multidrug-resistant clone [38]. In Ecuadorian isolates derived from the environment, this sequence type has been linked to integrons carrying resistance markers for aminoglycosides and trimethoprim [23]. In fact, our analysis confirmed the presence of these genes within this ST, in addition to genes conferring resistance to chloramphenicol. Moreover, isolates associated with both human and animal samples were identified. Notably, ST162 has been documented in such samples in Ecuador, Bolivia, and Colombia, although references to integrons have not been previously noted [39,40,41]. Similarly, our findings reveal that ST155, isolated from human, animal, and environmental sources, harbored integrons containing genes conferring resistance to the specified antibiotics. ST155 is another well-established multidrug-resistant clone [42] observed in Peru, Ecuador, and Colombia; however, previous studies have not associated it with integrons [43,44,45]. Interestingly, in Bolivia, this sequence type is associated with the presence of the *intI1* gene, although no references to gene cassettes within the variable region were identified [27]. ST58, ST744, ST90, and ST156 are recognized MDR clones [46,47,48,49] that have been documented in the area without previous references to integrons [27,40,50,51]. However, our investigation revealed that isolates belonging to these STs did indeed carry integrons containing resistance markers for aminoglycosides, trimethoprim, phenicols, and quinolones. Similarly, *E. coli* strains from the ST152, ST177, and ST449 clones also harbored integrons with the mentioned resistance markers. Although these clones are known for their multidrug resistance [52,53,54], they have not been previously documented in the area. 

The majority of pathogenic STs harbored integrons carrying markers for aminoglycosides and trimethoprim. Specifically, ST131, ST1193, ST38, and ST457 were exclusively associated with these genes. These sequence types, along with ST10, ST48, ST410, and ST69, are well known for their involvement in extraintestinal infections [55,56,57,58,59,60]. Furthermore, the latter four also carried genes conferring resistance to chloramphenicol, while ST10 additionally contained markers for β-lactams. Integron-containing ST10 and ST410 have been documented in Ecuador and Bolivia [23,24,27], whereas the remaining STs have not been directly linked to these platforms. In Bolivia, ST48, ST410, and ST69 isolates were found to be positive for the *intI1* gene and harbored many of the aforementioned resistance genes, although no references to the variable regions of integrons were identified [27]. These clones have also been reported in other countries, but the presence of integrons was not specifically assessed [10,45,51,61,62,63]. ST23, ST648, and ST354 have not been previously documented in the countries under study. However, these STs have been associated with extraintestinal infections [59,64,65]. Our analysis revealed that isolates belonging to these clones carried genes responsible for aminoglycosides, trimethoprim, and chloramphenicol resistance.

The most prevalent genes identified were those encoding various aminoglycoside adenylyl transferases (AadA) and dihydrofolate reductases (DfrA). These markers have previously been reported in *E. coli* associated with integrons in the scrutinized area, with specific alleles such as *dfrA1*, *dfrA5*, *dfrA7*, *dfrA12*, *dfrA15*, *dfrA17*, *aadA1*, *aadA2*, and *aadA5* documented [21,22,36]. In addition to these known sequences, our findings revealed the presence of previously unreported genes, including *aadA2b*, *aadA16*, *aadA17*, *aadA22*, *dfrA14*, *dfrA16*, and *dfrA27*. Furthermore, other markers for aminoglycosides, such as those encoding acetyl and nucleotidyl transferases, were found. While these genes have been described in *E. coli* in the area [22], they have not been directly linked to integrons. Resistance to chloramphenicol was attributed to acetyl transferases (Cat) and efflux pumps (Cml1), predominantly identified in animal and human samples. Specifically, genes encoding CatB3 have been detected in clinical isolates from Peru [21]. The sequences identified in our study have been documented in clinical and animal sources from Bolivia and Ecuador, although a direct relationship with integrons was not assessed [27,33].

Our findings show that resistance to β-lactams was associated with hydrolases encoded by *bla-OXA1* and *blaDHA4*, which were found in human and environmental isolates. A study reported the presence of *intI1* and *bla-OXA1* genes in both environmental and human isolates, although it was not specified whether *bla-OXA1* was located in the variable region [27]. However, in Colombia, a class 1 integron carrying the *blaVIM-4* gene, encoding for a metallo-β-lactamase, has been documented in clinical isolates [35]. Quinolone resistance was determined to be mediated by the *aac(6′)-Ib-cr* and *qnrB4* genes. These markers have been documented in numerous studies in the area, particularly in association with clinical and environmental samples, yet they have not been directly related to integrons [22,25,27].

Among the identified sequences, a total of 27 different gene cassette complexes were detected, all of which contained resistance genes. Integrons are commonly recognized as genetic structures with a low cost [66]; specifically, the number of gene cassettes has a notable impact on their fitness. Integrons carrying a larger number of gene cassettes tend to incur higher costs, leading to their decreased prevalence. Consequently, integrons harboring fewer, less costly cassettes are more commonly observed [67,68]. In fact, over 70% of predicted integrons were found to have one or two cassettes, and less than 1% contained more than five resistance genes in the variable region. Moreover, in half of the integrons, the first position was occupied by either an *aadA* or a *dfrA* gene. These cassettes contain highly recombinogenic *attC* sites and are frequently detected in such positions in class 1 integrons due to their low cost [66]. Additionally, the relatively costly *aac(6′)-lb* gene was found at the first position in approximately 8% of integrons. It has been suggested that this cassette must be located near the promoter to achieve proper expression levels [66].

Our results revealed that integrons are more predominantly present and diverse in the studied area than previously reported in the literature. Additionally, they carry a greater number of genes than previously documented. It is worth noting that only simple integrons containing two cassettes in their variable region have been reported in *E. coli*. The present results highlight that larger integrons containing more than two cassettes are prevalent among relevant STs. These complex integrons may play an important role in expanding the array of resistant markers found in *E. coli*. They carry genes conferring resistance not only to antibiotics commonly used as first-line defenses against *E. coli*—such as fluoroquinolones, trimethoprim–sulfamethoxazole, and cephalosporins—but also to aminoglycosides, which are typically reserved for treating serious infections [69,70]. Resistance markers against chloramphenicol, clindamycin, and rifampicin were also detected. These drugs may serve as viable alternatives when other antibiotics are ineffective or deemed inappropriate [71,72,73]. Certainly, integrons are extensively distributed among *E. coli* strains documented across diverse geographical regions [20], and they are thought to play a crucial role in the emergence of multidrug-resistant phenotypes within *Enterobacteriaceae* [16]. These mosaic structures act as reservoirs of exchangeable cassettes, which are not only linked to drug resistance but also to virulence and pathogenicity [74].

The dataset utilized in this study was constructed using *E. coli* sequences sourced from EnteroBase, potentially introducing bias toward isolates derived from culturable and pathogenic bacteria. While it is expected that novel isolates will continue to be reported, the ones examined in this study serve as relevant examples. Moreover, the approach employed in this study relies on a database of well-described integrons, which only represents a partial population. However, despite these limitations, the findings presented here contribute to advancing our understanding of the relationship between integrons, resistance genes, and *E. coli* in the Andean region.

## 4. Materials and Methods

### 4.1. Selection of Dataset

A dataset of *E. coli* isolates (n = 2533) that were whole-genome-sequenced was compiled from EnteroBase (http://enterobase.warwick.ac.uk/, accessed on 22 March 2023), a platform dedicated to studying genomic variation in enterobacteria. The compilation of this dataset adhered to specific criteria: (i) inclusion of isolates reported from countries within the Andean Community, including Ecuador, Colombia, Bolivia, and Peru, with the addition of data from Venezuela up to 2006, as it was a former member; (ii) collection of samples spanning the period 1993 to 2023; and (iii) selection of non-repetitive whole genomes. Sequences in FASTA format were downloaded on 22 March 2023. 

### 4.2. Multilocus Sequence Type Identification

The software MLST v2.19.0 developed by Torsten Seemann was employed to determine the sequence types (STs) using default settings (https://github.com/tseemann/mlst; accessed on 22 February 2023). The allele analysis scheme utilized for this purpose was from PubMLST.org [75]. The MLST scheme used was based on seven consensus genes, *adk*, *fumC*, *gyrB*, *icd*, *mdh*, *purA*, and *recA*.

### 4.3. IntFinder v1.0

IntFinder v1.0, developed by the Center for Genomic Epidemiology at the Technical University of Denmark (https://bitbucket.org/genomicepidemiology/intfinder/src/master/; accessed on 22 May 2023; software version: 2019-12-18; database version: 2019-11-29), was utilized to identify resistance integrons with modified parameters: threshold, 0.9, and min cov, 0.9. IntFinder v1.0 is accessible online (https://cge.food.dtu.dk/services/IntFinder-1.0/; accessed on 24 May 2023) and utilizes k-mer alignment for sequence detection, leveraging KMA v1.3.9, developed by the Center for Genomic Epidemiology at the Technical University of Denmark (https://bitbucket.org/genomicepidemiology/kma/src/master/, accessed on 24 May 2023) [76]. This alignment methodology enables detection based on an adjustable similarity threshold. Integron identification relies on detecting the presence of the integrase sequence, specifically, *intI1*. The integron database comprises data obtained from the public repository INTEGRALL (http://integrall.bio.ua.pt/; accessed on 24 May 2023) [77]. The database employs the unique numbers assigned by INTEGRALL to each integron (ln) [31,78]. Prediction of antimicrobial resistance genes in the integron database was carried out using the local standalone version of ResFinder v4.1 developed by the Center for Genomic Epidemiology at the Technical University of Denmark (https://bitbucket.org/genomicepidemiology/resfinder/src/master/; software version: 2019-01-29; database version: 2019-02-20, accessed on 24 May 2023) [79]. This analysis employed parameters requiring a minimum sequence identity of 90% and a minimum coverage of 60%. 

## 5. Conclusions

In this study, we investigated the occurrence and diversity of class 1 integrons in isolates reported from countries of the Andean Community using an in silico approach. Utilizing IntFinder v1.0, we found that almost one-third of the isolates tested positive for integrons. Class 1 integrons were identified in environmental, food, human, and animal isolates belonging to various relevant clones known for their pathogenicity or multidrug resistance. Overall, the integrons carried cassettes conferring resistance to antibiotics used in *E. coli* infections, such as fluoroquinolones, trimethoprim, cephalosporins, and aminoglycosides. The integrons identified in this study exhibited a greater number and variety of cassettes compared with what has been previously reported in the literature. Furthermore, the majority of integrons observed carried only two or three cassettes in their variable regions. However, in certain cases, four or even six cassettes were identified. These large mosaic structures harbor markers conferring resistance to various antibiotic classes, thereby aiding bacteria in adapting to environmental stress. In South American countries, several factors are recognized as favoring the spread of antibiotic-resistant bacteria. Therefore, monitoring the presence and diversity of these platforms in the region appears imperative. In silico studies are not only useful for identifying genetic traits associated with mobile elements but also contribute to recognizing emergent patterns of resistance, particularly in species inhabiting the intestine and regularly exposed to antibiotic pressure.

## Figures and Tables

**Table 1:** Resistance markers located in the variable regions of class 1 integrons detected in *E. coli* isolates reported in countries of the Andean Community.

Integron	Gene Cassettes in the Variable Region	Antimicrobial Resistance Pattern	Frequency (%)	Accession Number
In1741	*aadA1*, *cmlA1*, *aadA2*, *dfrA12*, *aadA17*, *lnu(F)*	AG, CHL, FA, LIN	0.4	CP042600
In37	*aac(6′)-Ib-cr*, *blaOXA-1*, *catB3*, *arr-3*	AG, CIP, PEN-CP, CHL, RIF	0.1	AY259086
In1021	*aac(6′)-Ib-cr*, *arr-3*, *dfrA27*, *aadA16*	AG, CIP, RIF, FA	0.1	KF921558
In1001	*aac(6′)-Ib3*, *aac(6′)-Ib-cr*, *catB3*, *dfrA1*	AG, CIP, CHL, FA	0.5	KF921553
In1598	*aadA16*, *dfrA27*, *arr-3*, *aac(6′)-Ib-cr*	AG, FA, RIF, CIP	0.4	MG196293
In1558	*dfrA12*, *aadA2*, *cmlA1*, *aadA1*	FA, AG, CHL	8.5	CP031549
In640	*dfrA12*, *aadA2*, *cmlA1*, *aadA1*	FA, AG, CHL	1.4	FM244708
In1632	*aadA1*, *cmlA1*, *aadA2b*	AG, CHL	2.2	CP034788
In1671	*aadA1*, *cmlA1*, *aadA2b*	AG, CHL	0.3	CP036168
In1405	*aadA22*, *lnu(F)*, *sul3*	AG, LIN, SUL	0.9	CP021843
In1004	*aadA2b*, *cmlA1*, *aadA1*	AG, CHL	10.0	KF921558
In1153	*aadA2b*, *cmlA1*, *aadA1*	AG, CHL	1.7	CP010575
In1179	*aadA2b*, *cmlA1*, *aadA1*	AG, CHL	0.3	CP011644
In1621	*blaDHA-1*, *qnrB4*, *dfrA17*	PEN-CP, CIP, FA	0.4	MK048477
In1058	*blaOXA-4*, *aadA2*, *cmlA1*	PEN-CP, AG, CHL	0.4	KJ463833
In1262	*aadA2*, *dfrA12*	AG, FA	0.3	KX710093
In322	*aadA1*, *blaOXA-1*	AG, PEN-CP	8.6	AM991977
In1265	*aadA1*, *dfrA1*	AG, FA	7.4	CP011540
In1077	*aadA1*, *aac(3)-VIa*	AG	2.3	CP009409
In1637	*aadA2*, *dfrA12*	AG, FA	0.1	LN830952
In1756	*aadA2*, *dfrA12*	AG, FA	0.1	CP042894
In1438	*aadA2*, *dfrA12*	AG, FA	14.3	CP022692
In406	*aadA2*, *dfrA12*	AG, FA	0.1	AP012055
In1546	*aadA5*, *dfrA17*	AG, FA	17.2	CP031110
In294	*ant(2″)-Ia*, *aadA2b*	AG	0.1	AJ971341
In1412	*dfrA12*, *aadA2*	AG, FA	0.4	CP019647
In1181	*dfrA17*, *aadA5*	AG, FA	0.1	CP006642
In1449	*dfrA17*, *aadA5*	AG, FA	0.3	CP023145
In1450	*dfrA17*, *aadA5*	AG, FA	2.7	CM008265
In1363	*lnu(F)*, *aadA17*	LIN, AG	0.5	CP019443
In1612	*dfrA5*	FA	3.3	CP034201
In862	*aadA1*	AG	0.8	CP011540
In530	*aadA1*	AG	3.9	AM055748
In18	*dfrA1*	FA	0.1	X17478
In191	*dfrA14*	FA	8.1	HF545433
In1210	*dfrA16*	FA	0.3	KT884517
In1205	*dfrA17*	FA	1.2	CP012626

AG: aminoglycosides; CHL: chloramphenicol; FA: folate antagonist; LIN: lincosamide; PEN: penicillin; CP: cephalosporin; RIF: rifampicin; SUL: sulfonamide.

## Data Availability

The data are available upon request.

## Supplementary Materials

The following supporting information can be downloaded at https://www.mdpi.com/article/10.3390/antibiotics13050394/s1: Table S1. Dataset of whole-genome-sequenced *E. coli* isolates in Andean Community. Table S2. Integron-positive isolates.
